# Determining extracellular vesicles properties and miRNA cargo variability in bovine milk from healthy cows and cows undergoing subclinical mastitis

**DOI:** 10.1186/s12864-022-08377-z

**Published:** 2022-03-07

**Authors:** Mara D. Saenz-de-Juano, Giulia Silvestrelli, Stefan Bauersachs, Susanne E. Ulbrich

**Affiliations:** 1 ETH Zurich, Animal Physiology, Institute of Agricultural Sciences, 8092 Zurich, Switzerland; 2 Institute of Veterinary Anatomy, Functional Genomics, University of Zurich, Eschikon 27, AgroVet-Strickhof, 8315 Lindau, ZH Switzerland

**Keywords:** Extracellular vesicles, Milk, microRNA, Subclinical mastitis, Small RNA-seq

## Abstract

**Background:**

Subclinical mastitis, the inflammation of the mammary gland lacking clinical symptoms, is one of the most prevalent and costly diseases in dairy farming worldwide. Milk microRNAs (miRNAs) encapsulated in extracellular vesicles (EVs) have been proposed as potential biomarkers of different mammary gland conditions, including subclinical mastitis. However, little is known about the robustness of EVs analysis regarding sampling time-point and natural infections. To estimate the reliability of EVs measurements in raw bovine milk, we first evaluated changes in EVs size and concentration using Tunable Resistive Pulse Sensing (TRPS) during three consecutive days of sampling. Then, we analysed daily differences in miRNA cargo using small RNA-seq. Finally, we compared milk EVs differences from naturally infected udder quarters with their healthy adjacent quarters and quarters from uninfected udders, respectively.

**Results:**

We found that the milk EV miRNA cargo was very stable over the course of three days regardless of the health status of the quarter, and that infected quarters did not induce relevant changes in milk EVs of adjacent healthy quarters. Chronic subclinical mastitis induced changes in milk EV miRNA cargo, but neither in EVs size nor concentration. We observed that the changes in immunoregulatory miRNAs in quarters with chronic subclinical mastitis were cow-individual, however, the most upregulated miRNA was bta-miR-223-3p across all individuals.

**Conclusions:**

Our results showed that the miRNA profile and particle size characteristics remained constant throughout consecutive days, suggesting that miRNAs packed in EVs are physiological state-specific. In addition, infected quarters were solely affected while adjacent healthy quarters remained unaffected. Finally, the cow-individual miRNA changes pointed towards infection-specific alterations.

**Supplementary Information:**

The online version contains supplementary material available at 10.1186/s12864-022-08377-z.

## Background

Mastitis, the inflammation of the mammary gland, is one of the most costly diseases in dairy farming [1]. It poses a major issue to combat as it is the main reason why antibiotics are used in dairy cattle. Cows with mastitis produce less milk of lower quality. Besides, mastitis decreases reproductive efficiency and adversely affects animal welfare [2]. The most common causes of mastitis are gram-positive and -negative bacteria that enter through the teat channel and establish in the mammary gland tissue. Furthermore, mastitis can also be developed after a viral, fungal, or protothecal infection [1]. Depending on the type of microorganism and the associated toxins, the response of the mammary gland varies. Gram-negative bacteria generally generate an acute immune response with clinical symptoms such as fever, udder damage or milk alterations (clinical mastitis) [1]. On the other hand, gram-positive bacteria trigger a moderate response that is asymptomatic (subclinical mastitis), but can ultimately result in chronic or life-long disease [1]. Unfortunately, the most frequent type of mastitis is the subclinical form, explaining why it can easily be transmitted to other animals in the herd during routine milking [3]. Subclinical mastitis is characterized by an endothelial cell malfunction that facilitates the unregulated accumulation of leukocytes at the site of infection, enhances leakage of plasma proteins into mammary tissues and disrupts the blood flow [4]. This inflammatory response results in an increase in immune cells in the milk indicated by a rise in the somatic cell count (SCC), which indicates the intramammary infection [5]. The optimal cut-off point to distinguish between infected and uninfected quarters has been established at 200′000 cells/ml [6, 7].

Biomarkers play a key role in defining and characterizing animal diseases [8]. MicroRNAs (miRNAs) are short non-coding RNA sequences (around 22 nucleotides) that have a critical function in posttranscriptional regulation of gene expression [9]. Since miRNA can circulate in body fluids and are dysregulated in a wide variety of diseases and syndromes, they have been proposed as non-invasive diagnostic biomarkers of disease status in human and livestock [10, 11]. In milk, it has been observed that miRNA profiles change upon mammary infection, lactation periods or breast cancer (reviewed by [12]). Thus, milk miRNAs have been proposed as diagnostic, prognostic, and predictive biomarkers of different mammary gland conditions. Recent technological advancements have made the visual detection of miRNA in raw milk possible by using RNA-functionalized gold nanoparticles [13].

In general, functional miRNAs circulate in body fluids associated with RNA-binding proteins or encapsulated in extracellular vesicles (EVs), lipid-rich vesicles or milk fat globules [12]. Since EV-delivered miRNA constitute a mechanism of regulating the inflammatory response [14], several studies have been conducted to evaluate the short-term changes in EV cargo due to mastitis [15, 16]. These experiments evidenced differences in EV cargo miRNAs upon inflammation, suggesting new potential biomarkers for early mastitis diagnosis. However, the latter studies were designed to induce the inflammation by inoculating a controlled amount of gram-positive *Staphylococcus aureus* (*S. aureus*) to a healthy quarter. Because it is known that subclinical mastitis is never produced by one unique pathogen only, the results might not adequately mimic the pathogenesis of the disease.

Up to now, milk EVs publications related to subclinical mastitis have used the same quarter as control quarters before the *S. aureus* challenge [16, 17], or uninfected quarters from different cows [15, 18, 19]. The central suspensory ligament and the fine membranes of the bovine mammary gland are physical barriers that separate the four quarters in tissue and blood supply [20]. Thus, cows can develop a local subclinical infection in only one of the quarters [7, 21]. This allowed within-udder experiments that include both treated and control quarters of a single cow, avoiding cow-bias [5, 20, 22]. Recent results, however, confirmed that the immune response to intramammary infection in a single mammary gland quarter alters the milk composition and the health status to the adjacent quarters [23]. Therefore, it is still unknown whether healthy adjacent quarters can be used as a control when evaluating milk EVs changes due to natural infections.

Daily fluctuation in SCC, milk composition and number of bacteria is normal in subclinical mastitis [24–26]. For that reason, the evaluation of several successive sampling is preferable to the interpretation of individual sampling [25]. In human breast milk, it has been reported that both secreted mRNA and miRNA might change throughout the day [27, 28]. However, to our knowledge, a rigorous evaluation of the daily stability of EV-miRNA to support its biomarker potential is still lacking.

We hypothesised that like other components in milk, EVs are dynamic and cow-dependent, and this needs to be taken into account when comparing healthy and subclinical mastitis, especially in naturally infected cases. Thus, the aims of this study were i) to estimate the daily and cow-individual milk EVs variability regarding size, concentration and miRNA cargo in health and subclinical mastitis; ii) to investigate milk EVs size, concentration and miRNA cargo between healthy quarters of inflamed and uninflamed udders and iii) to elucidate milk EVs alterations in chronic subclinical mastitis.

## Results

### Milk somatic cell count (SCC)

**Fig. 1:**
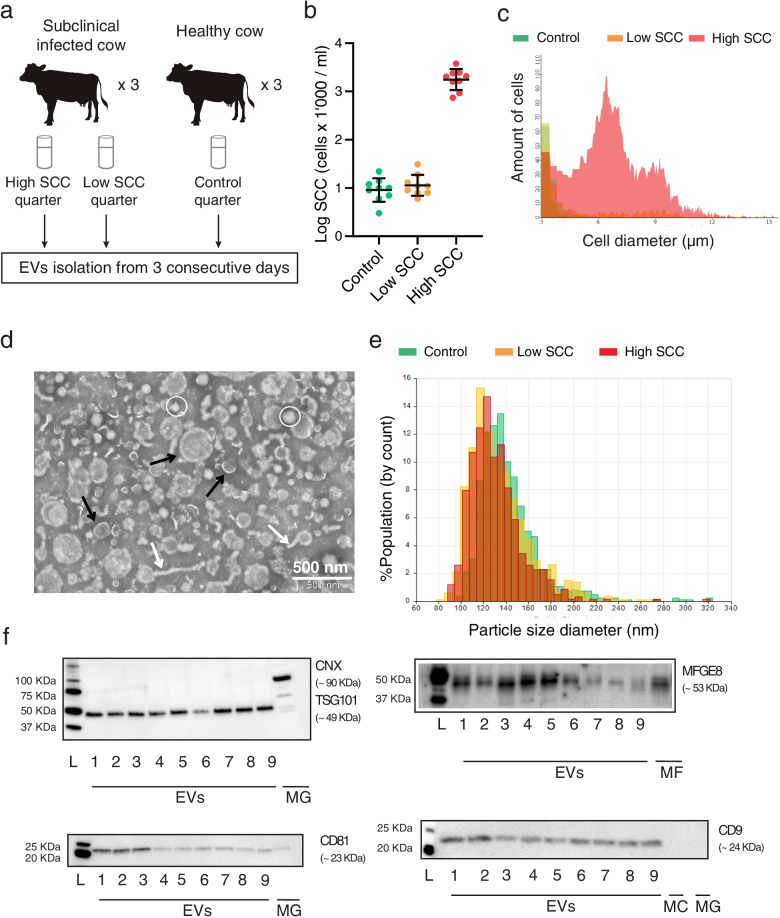
**a** Experimental design. EVs: Extracellular Vesicles **b** Scatterplot showing the somatic cell count (SCC) of the Control group, the Low SCC and the High SCC on a logarithmic scale. Lower-case letters denote significant differences (*P* < 0.05) after applying non-parametric Kruskal-Wallis and Dunn’s multiple comparisons tests. **c** Diameter distribution of milk cells in one High SCC (red), Low SCC (yellow) and Control (Green) sample, respectively. **d** Transmission electron microscopy (TEM) observation of isolated milk EVs. Black arrows indicate microvesicles and exosomes; white ovals highlight non-vesicles and white arrows point at shapeless aggregations with low electron density [29]. **e** Representative graphs of size distribution from three milk EVs samples measured with tunable resistive pulse sensing technology. **f** Western blot characterization for the EVs protein markers CXN, TSG101, CD81, CD9 and MFGE8. The absence of CXN suggests no contamination during the isolation with intracellular debris; L: Ladder; 1-9: Milk EVs pellets; MG: Mammary gland tissue; MF: Milk Fat; MC: Milk cells. Full-length blots are presented in Supplementary Fig. S4

Two groups of cows were defined regarding their SCC and California mastitis test (CMT) parameters: i) subclinically infected cows, showing a CMT positive and ii) healthy cows with a CMT negative test for at least two consecutive months. In healthy cows (Control), the SCC was lower than 50.000 cells/ml in all quarters, and one quarter was randomly selected as control (Fig. 1a). When the foremilk SCC is relatively low, the variation in SCC in the remaining milk is marginal, indicating that there is no ongoing infection [30]. From subclinically infected cows, two quarters were selected (Fig. 1a), one with a High SCC (> 400′000cells/ml) and another with a Low SCC (< 50′000cells/ml). To ensure the stability of the inflamed and healthy quarters, the milk SCC was determined for ten days in the morning and the afternoon milking. To study the milk EVs stability, we analysed the size, concentration and miRNA cargo from three consecutive days from the same cow and the same quarter.

SCC was stable in each cow and quarter along 10 days of milking (morning and afternoon) (Supplementary Table S1). From each quarter, three consecutive days were selected for EVs isolation and analysis. From the subclinically infected cows, Cow 4 had the lowest SCC average (1′219’000 cells/ml), while Cow 6 had the highest (2′074’000 cells/ml).

The SCC average of all High SCC quarters was 1′955’000 ± 900 cells/ml, while for the Low SCC and Control quarters the SCC average was 12′890 ± 8 cells/ml and 10′330 ± 5 cells/ml, respectively (Fig. 1b). Significant differences were found between High SCC and Low SCC (*P* < 0.0001) and between High SCC and Control (*P* < 0.0001). No significant differences were found between Low SCC and Control (*P* > 0.9). As Fig. 1c shows, High SCC milk (red) had a higher amount of cells between 5 and 9 μm of diameter, typical of lymphocytes [31], than Low SCC (yellow) and Control (green).

### Milk EVs isolation and characterization

Transmission electronic microscopy (TEM) confirmed the heterogeneity and integrity of EVs, showing populations of small and large vesicles resembling exosomes (30-110 nm) and microvesicles (> 100 nm), respectively (black arrows, Fig. 1d) [29]. We also observed the presence of some particles (white ovals, Fig. 1d) and shapeless aggregations with low electron density (white arrows, Fig. 1d) [29]. A lower amount of exosomes and microvesicles were obtained when using only differential ultracentrifugation (12′000 g, 35′000 g, 70′000 g and 100′000 g) and 0.25 M EDTA for casein precipitation as previously described [32] (Supplementary Fig. S1a-b). The size distribution analysis with TRPS showed that the particle size range was between 49 and 427 nm for High SCC, between 47 and 435 nm for Low SCC, and between 48 and 384 nm for Control (Supplementary Table S2). Figure 1e shows an example of a particle size histogram of one sample from each experimental group. The WB of EVs samples confirmed the presence of several EVs markers [33] (Fig. 1f). In particular, we detected the presence of the transmembrane proteins CD81 and CD9, and the cytosolic protein enriched in exosomes tumor susceptibility gene 101 (TSG101). Moreover, we also found the presence of milk fat globule-EGF factor 8 protein (MFGE8). The absence of Calnexin (CNX) suggested that there was no intracellular debris contamination during EVs isolation and that the majority part of EVs belonged to a small subtype population [33]. Without acid precipitation, the number of caseins left in the EVs pellet was similar to the one present in the skim milk (Supplementary Fig. S1c,d), which could explain why we observed the presence of TSG101 and MFGE8 protein (Supplementary Fig. S1e,f), but not CD81 (Supplementary Fig. S1g).

### RNA extraction

**Fig. 2:**
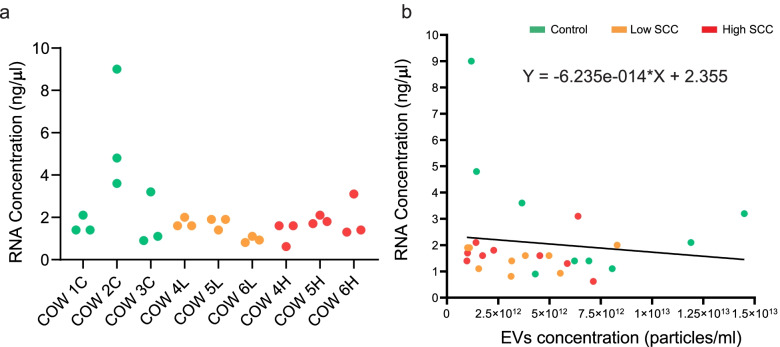
**a** RNA concentration of milk EVs in individual quarters. **b** Correlation between RNA and EVs concentration. Abbreviations: C: Control group, L: Low SCC group, H: High SCC group

Bioanalyzer RNA profiles and miRNA concentration for each tested protocol are shown in Supplementary Fig. S2. RNA degradation of longer fragments (such as ribosomal RNA) due to RNase treatment generated increased amounts of short RNA fragments (Supplementary Fig. S2). We selected the miRNeasy kit for RNA isolation because it extracted more RNA than the RNeasy Micro kit and the Qiazol methods (Supplementary Fig. S2). The concentration of extracted RNA ranged from 0.62 ng/μl to 9 ng/μl (Supplementary Table S2). The amounts obtained were similar between days (Fig. 2a), between experimental groups (Fig. 2a, *P* > 0.05) and had no significant correlation with milk EVs concentration (*P* > 0.05, Fig. 2b).

### Small RNA sequencing results

The sequencing of 27 libraries in two HiSeq 2500 lanes resulted in 10.1 to 23.2 million raw reads per library (16.2 ± 3.35, Supplementary Table S3). The raw reads were processed by removing low-quality sequences, too short sequences, adapters and PCR duplicates. Then, all unique sequences and read counts for all samples were joined into a count table and ~ 3,800,000 unique sequences were obtained. After CPM filtering, we obtained 10,313 unique sequences and 1771 successfully sequences mapped to the *Bos Taurus* miRBase database. To increase the biological relevance of our findings, differentially expression analysis was performed using the read counts of all isomiRs grouped for their corresponding mature miRNA. The isomiR clustering resulted in 140 unique miRNAs (Supplementary Table S4). All quarter types (High SCC, Low SCC and control) had the same top-ten most abundant milk EVs miRNA (Supplementary Table S5).

### Milk EVs variability

We did not observe significant differences in the EVs mean or mode size, and the coefficient of variation (CV) was 1.4-8.9% and 0.7-9.8%, respectively (Supplementary Table S2). On the contrary, the concentration of isolated EVs was more different between days, with a CV between 37.1 and 69.2% (Supplementary Table S2). Importantly, these results were independent of the EVs isolation session (*P* > 0.05, Supplementary Fig. S3a). Time-course analysis of miRNA profiles showed that there was no change in miRNA cargo throughout the three consecutive days (adjusted *P* > 0.05).

### Milk EVs cow-individual variability

**Fig. 3.**
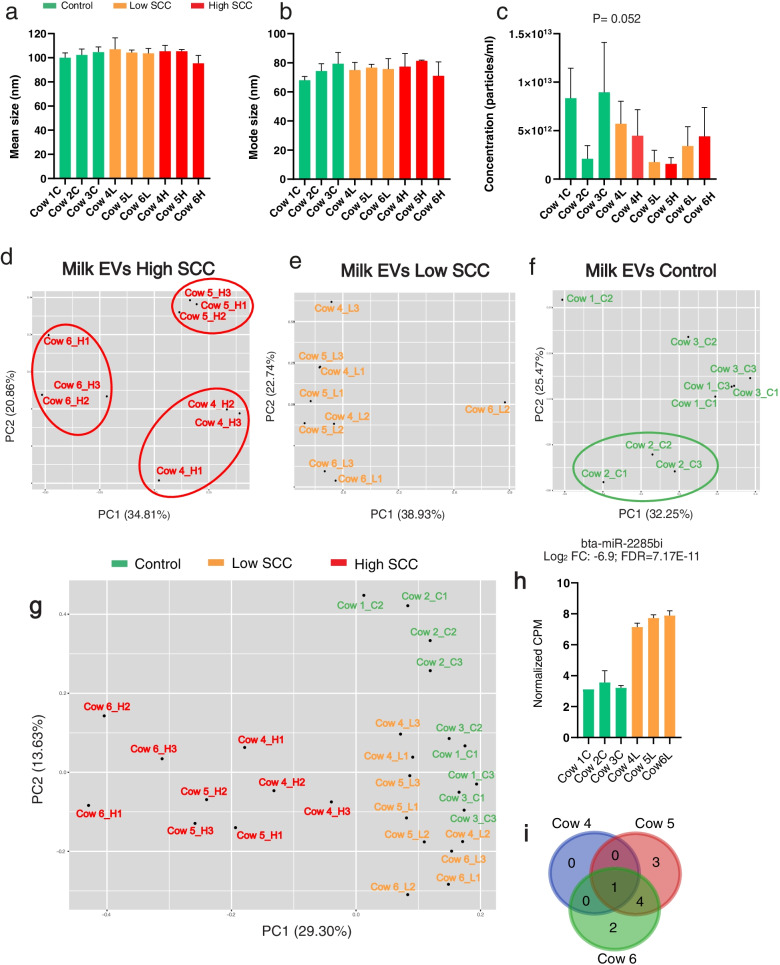
**a**-**c** Mean size, mode size and concentration of milk EVs in individual quarters. **d**-**f** Principal component analysis (PCA) of milk EVs miRNA (*n* = 122) in High SCC, Low SCC and Control. **g** PCA of milk EVs miRNA expression (*n* = 122) for all samples. **h** Normalized counts per million (CPM) for bta-miR-2285 t in Control (green) and Low SCC (orange). **i** Venn diagram showing the overlap of altered miRNAs between the three cows. Abbreviations: C: Control group, L: Low SCC group, H: High SCC group, the number indicates sampling day (1,2 and 3, respectively)

We did not observe significant differences in EVs mean or mode size between cows from the same or different experimental group (*P *> 0.05, Fig. 3a-b). On the other hand, we detected that the EVs concentration in milk seemed to be cow-dependent (*P* = 0.052, Fig. 3c). There was no significant correlation between isolated EVs and amounts of cells in the milk (*P* > 0.05, Supplementary Fig. S3b).

The principal component analysis (PCA) showed that the miRNA profile of High SCC quarters clustered very closely depending on the cow origin (Fig. 3d), while this was not the case for the Low SCC quarters from the same cows (Fig. 3e). On the other hand, PCA analyses from the Control quarters revealed that Cow 2 had a different miRNA profile than Cows 1 and 3 (Fig. 3f). While no significant differences were found between Cow 1 and 3, Cow 2 had 9 and 10 differentially expressed miRNAs compared to Cow 1 and Cow 3, respectively (FDR < 0.05, Supplementary Table S6).

### Minimal differences in miRNA EVs between low SCC and control milk samples

To assess whether an inflamed quarter induced changes in the miRNA of adjacent healthy quarters, we evaluated differences in miRNA between Low SCC and Control. The PCA analyses indicated that there was variation between High SCC and healthy quarters, while Low SCC and Control quarters clustered nicely together (Fig. 3 g). In Low SCC, we found a significant upregulation in bta-miR-2285bi (log2-fold change: 6.9, (Fig. 3 h) and significant downregulations in bta-miR-2285 t and bta-miR-2904 (log2-fold change: − 0.8 and − 1.5, respectively; FDR < 0.05).

### Cow-individual milk EV miRNAs in infected quarters

Significant miRNA alterations in High SCC quarters were different depending on the cow origin. In Cow 4 we observed that only bta-miR-223-3p was upregulated, while in Cows 5 and 6 we observed more differences (Table 1). Some miRNAs were equally altered in Cow 5 and 6 (Fig. 3i), with bta-miR-223-3p being the most altered in all the comparisons.

**Table 1:** List of differential miRNAs in milk EVs in High SCC and Low SCC from individual cows. FC: Fold change; FDR: False Discovery Rate

miRNA	Log2 FC	FDR	High SCC
Cow 4, High SCC vs. Low SCC			
bta-miR-223-3p	9.1	4.7E-02	↑
Cow 5, High SCC vs. Low SCC			
bta-miR-223-3p	8.1	5.5E-09	↑
bta-miR-142-5p	6.3	1.1E-05	↑
bta-miR-146b-5p	3.8	3.1E-09	↑
bta-miR-2284ab	2.3	3.7E-02	↑
bta-miR-2890	1.8	7.7E-03	↑
bta-miR-21-5p	0.8	4.1E-04	↑
bta-miR-93-5p	0.7	4.0E-02	↑
bta-miR-19b-3p	−2.4	2.1E-02	↓
Cow 6, High SCC vs. Low SCC			
bta-miR-223-3p	11.8	6.8E-03	↑
bta-miR-142-5p	10.3	7.4E-04	↑
bta-miR-2890	4.0	1.4E-03	↑
bta-miR-146b-5p	3.4	2.7E-04	↑
bta-miR-21-5p	1.3	1.7E-02	↑
bta-let-7i	1.3	4.1E-02	↑
bta-let-7d	1.3	1.7E-02	↑

### Milk EV alterations in subclinical mastitis after cow-bias correction

Milk EV in High SCC quarters showed different amounts for 18 miRNAs (FDR < 0.05, Table 2) compared to Low SCC quarters. Like before, the most upregulated miRNAs in High SCC were bta-miR-223-3p and bta-miR-142-5p, with 9.5 and 7.4 log2-fold change, respectively (Table 2). The most downregulated miRNA in High SCC was bta-miR-19b-3p.

**Table 2:** List of differential miRNAs in milk EVs from High SCC versus  Low SCC quarters. FC: Fold change; FDR: False Discovery Rate

High SCC vs. Low SCC			
miRNA	Log2 FC	FDR	High SCC
bta-miR-223-3p	9.5	6.1E-11	↑
bta-miR-142-5p	7.4	3.4E-09	↑
bta-miR-146b-5p	2.9	3.1E-07	↑
bta-miR-2890	2.4	1.5E-04	↑
bta-miR-2284ab	1.4	3.3E-02	↑
bta-miR-22-3p	0.7	1.8E-02	↑
bta-miR-21-5p	0.7	7.0E-03	↑
bta-miR-27b-3p	−0.4	1.8E-02	↓
bta-miR-181a-5p	−0.4	3.3E-02	↓
bta-miR-10,174-3p	−0.4	1.2E-02	↓
bta-miR-29a-3p	−0.6	1.2E-02	↓
bta-miR-29b-3p	−0.8	4.3E-02	↓
bta-miR-2285bf	−0.9	2.8E-02	↓
bta-miR-141-3p	−1.0	2.8E-02	↓
bta-miR-339a-5p	−1.1	3.0E-03	↓
bta-miR-374b-5p	−1.1	2.4E-02	↓
bta-miR-29c-3p	−1.2	2.0E-02	↓
bta-miR-19b-3p	−1.5	3.3E-02	↓

### Subclinical mastitis alters miRNAs related to the inflammatory system

**Fig. 4:**
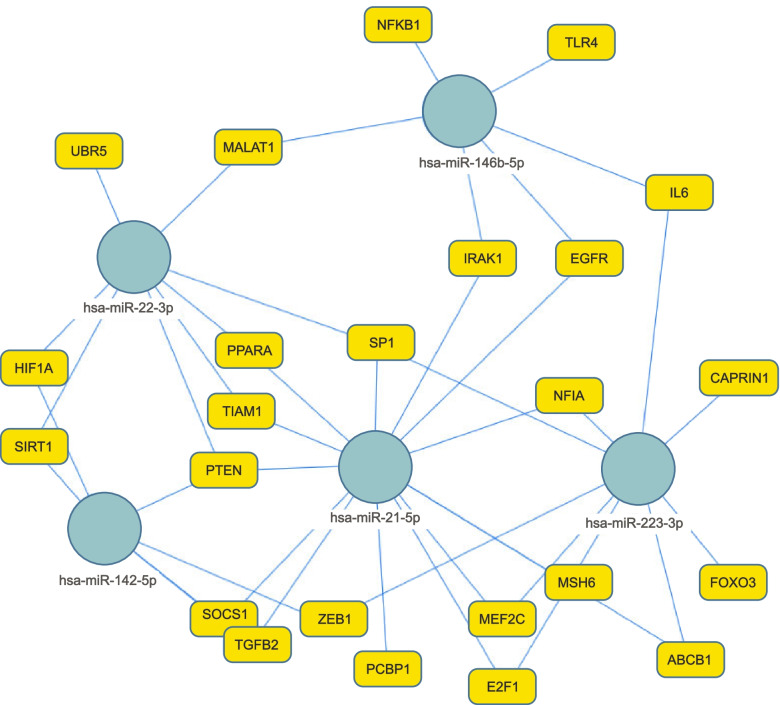
miRNA-target interaction network of upregulated miRNA in milk EVs in subclinical mastitis. Blue circles refer to miRNA, while yellow circles refer to their target genes

Based on miRTarbase, upregulated miRNAs in subclinical mastitis potentially target 80 genes, from which 48 were significantly targeted by two or more altered miRNAs (FDR < 0.05, Supplementary Table S7). The interactions between the different miRNAs and their target genes are shown in Fig. 4. The genes ATPase 13A3 (*ATP13A3*), BAG Cochaperone 2 (*BAG2*), Interleukin 6 (*IL6*), Peptidylprolyl Isomerase Domain And WD Repeat Containing 1 (*PPWD1*), RAS Related 2 (*RRAS2*), Sorting Nexin 24 (*SNX24*), Ubiquitin Specific Peptidase 48 (*USP48*) and Zinc finger E-box binding homeobox 1 (*ZEB1*) were targeted by two or more of the most commonly altered miRNAs bta-miR-223-3p, bta-miR-142, bta-miR-146b. DiANA-miRPath v3.0 based on TargetScan showed that there were 4 KEGG (Kyoto Encyclopedia of Genes and Genomes pathways [34, 35]) significantly altered, mainly involved in inflammatory reactions: NF-kappa B signalling pathway, Mucin type O-Glycan biosynthesis, Toll-like receptor signalling pathway and Cytokine-cytokine receptor interaction.

The downregulated miRNAs in subclinical mastitis potentially target 513 genes. From those, 205 genes were significantly targeted by three or more miRNAs. Specifically, the tumour suppressor phosphatase and tensin homolog (*PTEN*) gene was targeted by 6 downregulated miRNAs (Supplementary Table S8). Enrichment analysis of those genes showed that there were 691 KEGG pathways significantly altered (FDR < 0.05, Supplementary Table S8). Commonly altered pathways in miRTarbase and TargetScan were ECM-receptor interaction, amoebiasis, protein digestion and absorption, focal adhesion, gap junction, platelet activation, small cell lung cancer and PI3K-Akt signalling pathway.

## Discussion

The chronic subclinical mastitis is characterized by increased SCC and changes in milk composition for a prolonged time [36]. However, milk is a very dynamic fluid and its biologically active components, including miRNAs, change throughout the feeding period and day [28]. To evaluate the stability of milk EVs, we analysed their size, concentration and miRNA cargo during three consecutive days in health and subclinical mastitis. The time-course analysis showed that milk EVs mean size, mode size and miRNA profile were very stable between days, irrespective of the health status of the quarter. These result suggests that miRNAs packed in EVs are specific for the physiological state, and that sampling circumstances do not affect the overall results. In contrast, this was not the case for the EVs concentration. Most likely, the EVs extraction was not equally efficient for every sample, even if the isolation session did not affect the results. Nevertheless, despite the daily variability in EVs concentration, we observed a trend suggesting that EVs amounts in milk might be cow-dependent.

Several methods have been optimized to isolate EVs from raw or commercial milk [16, 19, 32, 37–42]. In our study, we decided to isolate EVs from skimmed milk by combining acid treatment, 0.22 μm filtering and ultracentrifugation [39]. Acid precipitation before ultracentrifugation allows purification of milk EV by removing casein micelles that have similar colloidal characteristics to EVs, and other milk whey proteins such as albumin, lactoferrin, and lactoglobulin [39, 40]. A later study also demonstrated that the use of acid generated purer EVs isolations than when only applying differential ultracentrifugation [43]. Despite the authors hypothesized that the proteins on the surface of EVs could be damaged, we could observe clear protein bands for known EVs surface markers in our EVs isolations, suggesting that the acid treatment was not affecting at least these proteins. Additionally, this protocol gave us better results on the protein profile and TEM than using only differential ultracentrifugation and EDTA for casein precipitation [32].

The thorough characterization of EVs was performed using TEM, TRPS and Western blot. The TEM showed that the use of one-step ultracentrifugation after 0.22 μm filtration generated a heterogeneous population of EVs (exosomes and microvesicles), non-EVs and protein aggregates. A density gradient separation and size exclusion chromatography (SEC) would have helped to eliminate low-density lipoproteins (LDL) and protein aggregates, respectively [40, 44]. However, it would have been difficult to separate high-density lipoproteins (HDL) and the addition of extra steps during isolation could have introduced more variability in the purified sample.

Recently, Herwijnen et al. [45] showed that milk EVs from different species have a similar miRNA profile, suggesting an evolutionary selection of miRNAs targeted to new-borns. In total, we identified 140 miRNAs, and we observed that all experimental groups including subclinical mastitis had the same top ten most abundant miRNAs, which were also included in the most common twenty milk EVs miRNAs listed in mammals. These results also agreed with milk EVs from commercial milk, specifically with the subset of EVs recovered after 12 K and 35 K ultracentrifugation [46]. Since before freezing, our milk samples were centrifuged at 3 K and 12 K to remove cells and cellular debris, we can assume that our EVs samples were not significantly contaminated with intracellular miRNAs.

Bovine milk whey contains indigenous RNases secreted by the mammary gland cells that can mediate an extracellular protective role [47, 48]. Milk miRNAs are unlikely to resist the high amount of RNases if not protected by protein complexes or vesicles [38]. It has been hypothesised that the majority of milk miRNAs are encapsulated within EVs to ensure their stability against the harsh environment in the digestive tract of the offspring [12, 38, 49]. We did not include an RNase treatment step because we observed that RNase treatment increased the amount of degraded RNA with similar fragment sizes as miRNAs. Moreover, it has been observed that specific miRNAs such as bta-miR-223-3p are affected by RNase digestion [38].

When we evaluated the EVs miRNAs in subclinical mastitis quarters, we observed clear differences between miRNA profiles depending on the cow-origin. This could mean that different cows reacted differently during chronic infection. On the other hand, we also saw that the more cells there were in the milk, the higher was the number of miRNA differences between healthy and infected quarters, which suggested that there might be a relation between the infection nature or progression and the miRNA content.

Despite the physical barriers, it is known that EVs can induce paracrine responses far from their origin [50]. To evaluate whether the inflammation of one quarter influences the EVs in milk from adjacent quarters, we compared healthy quarters from both inflamed and healthy udders. We saw that the overall differences between Low SCC and Control were minimal and that only bta-miR-2285bi was upregulated, and bta-miR-2285 t and bta-miR-2904 were slightly downregulated. Therefore, we concluded that healthy quarters within inflamed udders are good experimental controls in EV-research to avoid cow-bias. On the other hand, it has been reported that bta-miR-2285 t abundance changes during the different phases of lactation [51], and its downregulation has been linked to low milk productivity in beef cattle [52], but also to *S. aureus* infection [53]. Our results are in line with the assumption that infection in a single mammary gland quarter alters milk production in the adjacent quarters. Thus, further experiments should evaluate whether these changes in healthy adjacent quarters are common for chronic infections and present in earlier stages.

A dysregulation of expression levels of miRNAs can lead to chronic infections and inflammatory diseases [54]. In our study, a total of 18 miRNAs were found differentially expressed between inflamed and healthy quarters. The most differentially expressed miRNAs were bta-miR-223-3p, bta-miR-142-5p and bta-miR-146b-5p. Similar results were obtained 48 h after healthy quarters were challenged with a controlled amount of *S. aureus* [15, 16, 55, 56], with bta-miR-223-3p appearing as the most upregulated miRNA [15, 16]. For that reason, future research on milk biomarkers for health management should be devoted to this specific miRNA. MiR-223 has key roles in inflammation and infection, and it is deregulated in many different pathologies [57–60].

It has been shown that EVs from commercial milk contain detectable amounts of bta-miR-223, mostly in the 12 K and 35 K EV subsets [46]. This was not the case for our Low SCC and Control samples. This may be explained because commercial milk is composed of a pool of milk from different cows, on average displaying a higher SCC than in our Low SCC and Control cows. Benmoussa et al. (2019) demonstrated that human cells can take up functional bta-miR-223 from commercial milk EVs which can participate in the gene regulatory system of the recipient cells [46]. It is known that thermic conditions of pasteurization are not sufficient to eliminate bioactive milk EV, and there is rising concern that continuous exposure to milk miRNAs may confer substantial risk for the development of chronic diseases, including obesity, type 2 diabetes mellitus, osteoporosis and some common cancers (reviewed by [61]). In this sense, new technologies to easily detect miRNAs in milk, like the one developed by Sánchez-Visedo et al., 2020 [13], might be advantageous to both health management and food testing.

We did not find a significant correlation between the amounts of cells in milk and the EVs concentration, suggesting that the majority of milk EVs might be released from MEC in the alveoli. In agreement, it has been shown that the majority of miRNAs contained in milk derives from MEC and that there might be a specific selection of secreted miRNAs [46]. We compared our set of altered miRNAs to previously published data on in vitro culture of bovine primary MEC [62] as well as bovine immune cells [63–65]. While bta-miR-223-3p, bta-miR-142-5p, bta-miR-339a-5p, bta-miR-2890 and bta-miR2284ab were reported in immune cells; other altered miRNAs, including bta-miR-181a-5p, bta-miR-19b-3p, bta-miR-27b-3p, bta-miR-374b-5p, bta-miR-21-5p, bta-miR-146b-5p, bta-miR-29a-3p, bta-miR-29b-3p and bta-miR-29c-3p could have been released by either MEC and/or immune cells.

The increase of immune cells in milk from inflamed quarters can also explain the higher amount of bta-miR-223-3p, and that the cow with higher SCC contains the higher amount of this miRNA. Indeed, mammary gland epithelial cells from the cell line MAC-T did not express higher amounts of bta-miR-223-3p after *S. aureus* challenge [66]. This finding is also in line with previous studies in which the upregulation of bta-miR-223-3p was observed in mammary gland tissue biopsies after *S. aureus* [53] and *Streptococcus Uberis* infections [55].

## Conclusions

In conclusion, chronic subclinical mastitis induced changes in milk EVs miRNA cargo, but neither in EVs size nor concentration. Thus, milk EVs miRNA profiling provides a powerful tool to get new insights into the molecular background of subclinical mastitis physiology. While the miRNA profile and particle size characteristics remained constant throughout consecutive days, the EVs concentration was dependent on the individual cow and was highly variable. Extracellular vesicle miRNA alterations in chronic subclinical mastitis correlated to early post-infection, suggesting that for example, bta-miR-223-3p might be a potential indicator of subclinical mastitis progression and chronicity.

## Methods

### Animals

The experiment was performed with the experimental dairy cow herd at AgroVet-Strickhof, Lindau, Switzerland. Selected dairy cows (*n* = 6) were in mid-lactation [DIM = 126.3 ± 16.76; mean ± SEM] and had between one and three parities. All cows were producing > 25 kg of milk/day (mean milk yield ± SEM = 32.15 ± 1.7 kg). Each experimental group consisted of two Holstein cows and one Swiss Brown cow.

### Milk collection and SCC measurement

After discarding the first 5 ml, 50 ml of milk were manually collected during ten consecutive days from all individual quarters before the morning and the afternoon milking routine. Immediately, the somatic cell count (SCC) was determined using the DCC DeLaval machine (DeLaval). From selected samples, the milk cells diameter was determined with the Scepter™ 2.0 Cell Counter and the 40 μm aperture sensor (Merck). Before freezing, the milk fat and cell debris were removed after two centrifugation rounds at 3′000 *g* for 15 min at 4 °C and 12′000 *g* for 20 min at 4 °C. The skimmed milk was stored at − 20 °C until further use.

### Extracellular vesicles isolation

For each quarter (High SCC, Low SCC and Control), three skim milk samples from three consecutive days (24 h difference between milking) were selected for EVs isolation. Extracellular vesicles were isolated by combining acid treatment, 0.22 μm filtering and ultracentrifugation as previously described [39] (Fig. 1C). Briefly, 25 mL of skim milk were heated at 37 °C for 10 min. Then, to precipitate the casein micelles and other proteins, 1% of acetic acid (Sigma) was added and samples were centrifuged at 10′000 *g* for 10 min at 4 °C. The supernatant was filtered through 0.22 μm filter and samples were ultracentrifuged at 210′000 *g* for 70 min at 4 °C (Optimax 90XE, Beckman Coulter). The pellet was washed with PBS and ultracentrifuged again at 210′000 *g* for 70 min at 4 °C. Finally, the pellet was resuspended with 500 μl of PBS and centrifuged at 10′000 *g* for 5 min at 4 °C. The supernatant containing the EVs was carefully collected, divided into aliquots for further analysis and stored at − 80 °C until use.

### Extracellular vesicles characterization

#### Transmission electron microscopy (TEM)

Extracellular vesicles visualization was performed by the Scientific Center for Optical and Electron Microscopy (ScopEM) service of ETH Zurich. Briefly, three microliters of the vortexed dispersion were placed on glow discharged carbon-coated grids (Quantifoil, D) for 1 min. Negative contrast staining was done in 2% sodium phosphotungstate pH 7.2 for 1 s, followed by a second step for 15 s. Excess moisture was drained with filter paper and the imaging of the air-dried grids was done in a TEM Morgagni 268 (Thermo Fisher) operated at 100 kV. For each experimental groups, two replicates were analysed.

#### Protein extraction and Western blotting

Extracellular vesicle lysis and protein extraction was performed using Radioimmunoprecipitation assay buffer (RIPA buffer, Thermo Scientific) combined with 100X anti-protease cocktail (Thermo Fisher). Then, EV protein content was measured using the BCA high range assay kit (Thermo Fischer) and the Nanodrop 2000 (Thermo Fisher).

Western blots were performed by mixing 10 μg of protein with 5 μL Laemmli Buffer (Bio-Rad). When reductant conditions were necessary, 10% β-Mercaptoethanol was also added before 10 min incubation at 95 °C. Samples were loaded onto a 4-20% Mini-Protein TGX Stain-free Precast gel (Bio-Rad). Before transfer, stain-free gels were UV-activated using the ChemiDoc™ MP (Bio-Rad), and a picture from the loaded protein in the gel was taken for later normalization. Then, proteins were transferred onto a 0.2 μm PVDF trans-blot turbo transfer pack (Bio-Rad) using the Turbotransfer (Bio-Rad) and 1.3A, 25 V and 7 min as transfer conditions. Immediately, the membrane was blocked with TBST (Bio-Rad, 0.05% Tween 20) with 5% skim milk powder at room temperature for 1 h. Afterwards, the membrane was incubated overnight with the primary antibodies. The antibodies used were rabbit anti-TSG101 (Thermo Fisher, PA531260, 1:1′000), rabbit anti-Calnexin (Abcam, ab75801, 1:2′000), mouse anti-CD81 (Santa Cruz, sc166029, 1:300) and rabbit anti-MFGE8 (Sigma, HPA002807, 1:500). For CD81 and CD9 non-reductant conditions were used for the protein electrophoresis. Next day, the membrane was washed and incubated with the secondary antibodies goat anti-rabbit IgG-HRF (Santa Cruz Biotechnology, sc2004, 1:10′000) and goat anti-mouse IgM-HRF (Santa Cruz Biotechnology, sc2005 1:10′000). To visualise the latter, Precision Protein Strep Tactin-HRP was added as well (1:10′000, Bio-Rad). Finally, ClarityTM ECL substrate (Bio-Rad) was loaded onto the membrane and bands were visualized with ChemiDocTM MP. Mammary gland tissue (MG) and milk fat (MF) were used as a positive control for Calnexin and MFGE8, respectively.

#### Tunable resistive pulse sensing (TRPS)

Particle concentration and size distribution were measured using the qNano Gold system (Izon Science) and an NP150 Nanopore (Izon Science). Extracellular vesicles samples were diluted 1:100 in filtered PBS and CPC100 beads (Izon Science) were used as the calibration standard. Particles were measured using 46.0 mm stretch with a voltage of 0.6-1.4 V and a pressure between 4.46 and 7.33 mbar. The number of particles analysed per sample was at least 1000. The blockade magnitude of the calibration particles was above 0.2 nA and a new Nanopore was used in every different measurement day. Data were processed with Izon Control Suite software version 3.3 (Izon Science).

### Statistical analysis

Differences in particle concentration, diameter, and size distribution were determined by non-parametric Kruskal-Wallis test and Dunn’s multiple comparisons tests using GraphPad Prism version 8.2. Differences were considered significant when *P* < 0.05. Unless stated, all numbers are given as mean ± standard deviation (SD).

### Small RNA library preparation

A pool of EVs from several milk samples was generated to select the best method for small RNA extraction. Three different methods including RNeasy Micro kit (Qiagen), miRNeasy Micro Kit (Qiagen) and QIAzol (Qiagen) were tested with 200 μL of pooled milk EV and according to the manufacturer’s protocols. All methods were performed either with or without RNase A treatment (Qiagen, final concentration 0.5 μg/μL), consisting of 2 min incubation at room temperature before the addition of Lysis buffer or QIAzol. The presence of small RNA was confirmed and evaluated using Agilent Pico Kit and Agilent Small RNA kit (Agilent Technologies). We observed that RNA degradation of longer fragments (such as ribosomal RNA) after RNase treatment generated increased amounts of short RNA fragments (see results). The miRNeasy Micro Kit protocol without RNase treatment was thus selected for the EV samples included in this study. After small RNA extraction, RNA quantity was determined with Quantus™ Fluorometer and the QuantiFluor® RNA System kit (Promega). For each sample, 8.5 ng of RNA was used to generate the small RNA-seq libraries. Libraries were prepared using the NEXTflex™ Small RNA-Seq Kit v3 (Bioo Scientific) and were sequenced as one pool of 27 barcode-tagged samples on an Illumina HiSeq 2500 (126 bp single-end reads) on two lanes. Sequencing of the small RNA libraries was conducted at the Functional Genomic Center Zurich (FGCZ), and the resulting FastQ files were uploaded to our local Galaxy server installation [67].

### Small RNA-seq data analysis

Data analysis was performed on a local Galaxy system using an in-house developed pipeline with minor modifications [68, 69]. First, reads were trimmed using Trim Galore (Version 0.4.3 by Felix Krueger). With this tool, we removed the 3′ adapter sequence (TGGAATTCTCGGGTGCCAAGG) together with low-quality end reads (Quality score threshold = 30) and short reads (< 25 nucleotides). Then, Fastq files were quality checked with FastQC (Version 0.11.2) to control that the processing was done correctly. PCR duplicates were detected due to the four random nucleotides introduced with the adapters on each side of the RNA fragment and then removed using the tool “Collapse” (FASTX-toolkit by Assaf Gordon). Like that, we generated a count table for all obtained unique sequences and corrected for PCR duplicates. To remove sequences with negligible counts, sequencing errors and sequences with very low evidence for potential expression, the count table was filtered using the counts per million (CPM) filter. Using an in-house tool, the mean library size was calculated, and the CPM cut-off was applied at 6.66, corresponding to an average of > 10 reads per sample for at least 8 out of 27 libraries. Sequences were annotated using BLAST (Basic Local Alignment Search Tool) and “blastn-short” (Version 2.2.31), a tool optimized for sequences shorter than 50 bases. Alignments were performed against the miRBase database (Version 22.1, [70]) for mature miRNAs in *Bos taurus*. Only alignments without any mismatch to the canonical form in miRbase were included in further analysis. Finally, all isomiRs were grouped using the in-house tool “Group on data”, that summed the read counts of every miRNA with the same name. Differential expression analysis was performed using the Bioconductor package EdgeR [71]. Normalization of the read count data was done using TMM normalization [72] and GLM robust (estimateGLMRobustDisp) [73] included in EdgeR. The following comparisons were run: (a) Low SCC vs. Control; and (b) High SCC vs. Low SCC, including cow origin as a batch effect. Significant differences were considered when the adjusted P (false discovery rate, FDR) was < 5%. To evaluate whether miRNAs changed through the different sampling days a specific software for time-course analysis, ImpulseDE2 [74], was used including cow origin as a batch effect.

### Functional enrichment analysis

To understand the role of highly altered miRNAs during chronic subclinical mastitis, gene target, network and functional enrichment analysis were performed using MIENTURNET (MicroRNA ENrichment TURned NETwork) based on miRTarBase with human orthologues [75]. For the miRNA-target enrichment and network analysis, default parameters were applied, and results were considered as significant when FDR was < 0.05. Results were broadened with DiANA-miRPath v3.0 [76] and TargetScan Database [77] using FDR correction and conservative stats.

## Supplementary Information

**Additional file 1: Supplementary Figure S1-S5.**

**Additional file 2: Supplementary Table S1.** Somatic Cell Count in milk during the animal trial.

**Additional file 3: Supplementary Table S2.** Mean size, mode size, concentration of particles and RNA concentration in each EV sample.

**Additional file 4: Supplementary Table S3.** Sequencing output smallRNA-seq.

**Additional file 5: Supplementary Table S4.** All miRNAs found in milk EV samples.

**Additional file 6: Supplementary Table S5.** Top-ten most abundant miRNA in Control, Low SCC and High SCC samples.

**Additional file 7: Supplementary Table S6.** List of differential miRNAs between Control quarters of different cows.

**Additional file 8: Supplementary Table S7.** miRNA-target enrichment and network analysis of upregulated miRNAs.

**Additional file 9: Supplementary Table S8.** miRNA-target enrichment and network analysis of downregulated miRNAs.

## Data Availability

Small RNA-seq data have been deposited in the Gene Expression Omnibus database (GEO) under accession code GSE149856. All relevant data of our experiments have been submitted to the EV-TRACK knowledgebase (EV-TRACK ID: EV200027).

## References

[1] Wellnitz O, Bruckmaier RM (2012). The innate immune response of the bovine mammary gland to bacterial infection. Vet J.

[2] Duarte CM, Freitas PP, Bexiga R (2015). Technological advances in bovine mastitis diagnosis: an overview. J Vet Diagn Investig.

[3] Pumipuntu N, Kulpeanprasit S, Santajit S, Tunyong W, Kong-Ngoen T, Hinthong W, Indrawattana N. Screening method for Staphylococcus aureus identification in subclinical bovine mastitis from dairy farms. Vet World. 2017;10(7):721–6. 10.14202/vetworld.2017.721-726. 10.14202/vetworld.2017.721-726 PMC5553136 28831211

[4] Ryman VE, Packiriswamy N, Sordillo LM. Role of endothelial cells in bovine mammary gland health and disease. Anim Health Res Rev. 2015;16(2):135–49. 10.1017/S1466252315000158. 10.1017/S1466252315000158 26303748

[5] Wall SK, Wellnitz O, Bruckmaier RM, Schwarz D (2018). Differential somatic cell count in milk before, during, and after lipopolysaccharide- and lipoteichoic-acid-induced mastitis in dairy cows. J Dairy Sci.

[6] Gonçalves JL, Cue RI, Botaro BG, Horst JA, Valloto AA, Santos MV (2018). Milk losses associated with somatic cell counts by parity and stage of lactation. J Dairy Sci.

[7] Schepers AJ, Lam TJGM, Schukken YH, Wilmink JBM, Hanekamp WJA (1997). Estimation of variance components for somatic cell counts to determine thresholds for uninfected quarters. J Dairy Sci.

[8] Moore RE, Kirwan J, Doherty MK, Whitfield PD (2007). Biomarker discovery in animal health and disease: the application of post-genomic technologies. Biomark Insights.

[9] Kim VN (2005). MicroRNA biogenesis: coordinated cropping and dicing. Nat Rev Mol Cell Biol.

[10] Dong H, Gao Q, Peng X, Sun Y, Han T, Zhao B (2017). Circulating MicroRNAs as potential biomarkers for veterinary infectious diseases. Front Vet Sci.

[11] Tian F, Zhang S, Liu C, Han Z, Liu Y, Deng J (2021). Protein analysis of extracellular vesicles to monitor and predict therapeutic response in metastatic breast cancer. Nat Commun.

[12] Benmoussa A, Provost P (2019). Milk MicroRNAs in health and disease. Compr Rev Food Sci Food Saf.

[13] Sánchez-Visedo A, Gallego B, Royo LJ, Soldado A, Valledor M, Ferrero FJ, Campo JC, Costa-Fernández JM, Fernández-Argüelles MT. Visual detection of microRNA146a by using RNA-functionalized gold nanoparticles. Mikrochim Acta. 2020;187(3):192. 10.1007/s00604-020-4148-4. 10.1007/s00604-020-4148-4 32124045

[14] Alexander M, Hu R, Runtsch MC, Kagele DA, Mosbruger TL, Tolmachova T (2015). Exosome-delivered microRNAs modulate the inflammatory response to endotoxin. Nat Commun.

[15] Cai M, He H, Jia X, Chen S, Wang J, Shi Y, Liu B, Xiao W, Lai S. Genome-wide microRNA profiling of bovine milk-derived exosomes infected with Staphylococcus aureus. Cell Stress Chaperones. 2018;23(4):663-672. 10.1007/s12192-018-0876-3. 10.1007/s12192-018-0876-3 PMC6045547 29383581

[16] Sun J, Aswath K, Schroeder SG, Lippolis JD, Reinhardt TA, Sonstegard TS. MicroRNA expression profiles of bovine milk exosomes in response to Staphylococcus aureus infection. BMC Genomics. 2015:1–10. 10.1186/s12864-015-2044-9. 10.1186/s12864-015-2044-9 PMC4609085 26475455

[17] Reinhardt TA, Sacco RE, Nonnecke BJ, Lippolis JD (2013). Bovine milk proteome: quantitative changes in normal milk exosomes, milk fat globule membranes and whey proteomes resulting from Staphylococcus aureus mastitis. J Proteome.

[18] Ma S, Tong C, Ibeagha-Awemu EM, Zhao X (2019). Identification and characterization of differentially expressed exosomal microRNAs in bovine milk infected with Staphylococcus aureus. BMC Genomics.

[19] Ma S, Niu M, Hao Z, Liu M, Tong C, Zhao X (2021). Selective packaged circular RNAs in milk extracellular vesicles during Staphylococcus aureus infection may have potential against bacterial infection. RNA Biol.

[20] Nickerson, S.C. and Akers, R.M. 2011. Mammary gland, Encyclopedia of dairy sciences. 2nd edition. 3: 328-337. 10.1016/B978-0-12-374407-4.00290-9.

[21] Postle DS, Roguinsky M, Poutrel B (1978). Induced staphylococcal infections in the bovine mammary gland. Am J Vet Res.

[22] Wellnitz O, Arnold ET, Bruckmaier RM (2011). Lipopolysaccharide and lipoteichoic acid induce different immune responses in the bovine mammary gland. J Dairy Sci.

[23] Paixão MG, Abreu LR, Richert R, Ruegg PL (2017). Milk composition and health status from mammary gland quarters adjacent to glands affected with naturally occurring clinical mastitis. J Dairy Sci.

[24] Quist MA, LeBlanc SJ, Hand KJ, Lazenby D, Miglior F, Kelton DF (2008). Milking-to-milking variability for milk yield, fat and protein percentage, and somatic cell count. J Dairy Sci.

[25] Dohoo IR, Meek AH (1982). Somatic cell counts in bovine milk. Can Vet J.

[26] Sears PM, Smith BS, English PB, Herer PS, Gonzalez RN (1990). Shedding pattern of Staphylococcus aureus from bovine Intramammary infections. J Dairy Sci.

[27] Maningat PD, Sen P, Rijnkels M, Sunehag AL, Hadsell DL, Bray M (2009). Gene expression in the human mammary epithelium during lactation: the milk fat globule transcriptome. Physiol Genomics.

[28] Floris I, Billard H, Boquien CY, Joram-Gauvard E, Simon L, Legrand A (2015). MiRNA analysis by quantitative PCR in preterm human breast milk reveals daily fluctuations of hsa-miR-16-5p. PLoS One.

[29] Sedykh SE, Burkova EE, Purvinsh L, Klemeshova DA, Ryabchikova EI, Nevinsky GA. Milk Exosomes: isolation, biochemistry, morphology, and perspectives of use: IntechOpen; 2019. p. 28. Available from: https://www.intechopen.com/books/advanced-biometric-technologies/liveness-detection-in-biometrics

[30] Wellnitz O, Doherr MG, Woloszyn M, Bruckmaier RM (2009). Prediction of total quarter milk somatic cell counts based on foremilk sampling. J Dairy Res.

[31] Ruban GI, Kosmacheva SM, Goncharova NV, Van BD, Loiko VA (2007). Investigation of morphometric parameters for granulocytes and lymphocytes as applied to a solution of direct and inverse light-scattering problems. J Biomed Opt.

[32] Vaswani K, Koh YQ, Almughlliq FB, Peiris HN, Mitchell MD (2017). A method for the isolation and enrichment of purified bovine milk exosomes. Reprod Biol.

[33] Théry C, Witwer KW, Aikawa E, Alcaraz MJ, Anderson JD, Andriantsitohaina R, et al. Minimal information for studies of extracellular vesicles 2018 (MISEV2018): a position statement of the International Society for Extracellular Vesicles and update of the MISEV2014 guidelines. J Extracell Vesicles. 2018;7(1):1535750. 10.1080/20013078.2018.1535750. 10.1080/20013078.2018.1535750 PMC6322352 30637094

[34] Kanehisa M, Furumichi M, Sato Y, Ishiguro-Watanabe M, Tanabe M (2021). KEGG: integrating viruses and cellular organisms. Nucleic Acids Res.

[35] Kanehisa M, Goto S (2000). KEGG: Kyoto encyclopedia of genes and genomes. Nucleic Acids Res.

[36] Kirsanova E, Heringstad B, Lewandowska-Sabat A, Olsaker I (2019). Alternative subclinical mastitis traits for genetic evaluation in dairy cattle. J Dairy Sci.

[37] Blans K, Hansen MS, Sørensen L V., Hvam ML, Howard K a., Möller A, et al. Pellet-free isolation of human and bovine milk extracellular vesicles by size-exclusion chromatography. J Extracell Vesicles 2017;6(1). 10.1080/20013078.2017.1294340. 10.1080/20013078.2017.1294340 PMC5373680 28386391

[38] Benmoussa A, Ly S, Shan ST, Laugier J, Boilard E, Gilbert C, et al. A subset of extracellular vesicles carries the bulk of microRNAs in commercial dairy cow’s milk. J Extracell Vesicles. 2017;6(1). 10.1080/20013078.2017.1401897. 10.1080/20013078.2017.1401897 PMC5994974 29904572

[39] Somiya M, Yoshioka Y, Ochiya T. Biocompatibility of highly purified bovine milk-derived extracellular vesicles. J Extracell Vesicles. 2018;7(1). 10.1080/20013078.2018.1440132. 10.1080/20013078.2018.1440132 PMC5827637 29511463

[40] Morozumi M, Izumi H, Shimizu T, Takeda Y (2021). Comparison of isolation methods using commercially available kits for obtaining extracellular vesicles from cow milk. J Dairy Sci.

[41] Mukhopadhya A, Santoro J, Moran B, Useckaite Z, O'Driscoll L. Optimisation and comparison of orthogonal methods for separation and characterisation of extracellular vesicles to investigate how representative infant milk formula is of milk. Food Chem. 2021;353:129309. 10.1016/j.foodchem.2021.129309. 10.1016/j.foodchem.2021.129309 33725545

[42] Wijenayake S, Eisha S, Tawhidi Z, Pitino MA, Steele MA, Fleming AS (2021). Comparison of methods for pre-processing, exosome isolation, and RNA extraction in unpasteurized bovine and human milk. PLoS One.

[43] Rahman MM, Shimizu K, Yamauchi M, Takase H, Ugawa S, Okada A (2019). Acidification effects on isolation of extracellular vesicles from bovine milk. PLoS One.

[44] Brennan K, Martin K, FitzGerald SP, O’Sullivan J, Wu Y, Blanco A (2020). A comparison of methods for the isolation and separation of extracellular vesicles from protein and lipid particles in human serum. Sci Rep.

[45] van Herwijnen MJC, Driedonks TAP, Snoek BL, Kroon AMT, Kleinjan M, Jorritsma R (2018). Abundantly present miRNAs in Milk-derived extracellular vesicles are conserved between mammals. Front Nutr.

[46] Benmoussa A, Laugier J, Beauparlant CJ, Lambert M, Droit A, Provost P. Complexity of the microRNA transcriptome of cow milk and milk-derived extracellular vesicles isolated via differential ultracentrifugation. J Dairy Sci. 2019. 10.3168/jds.2019-16880. 10.3168/jds.2019-16880 31677838

[47] Ibuki F, Mori T, Matsushita S, Hata T (1965). Ribonuclease in bovine milk. Agric Biol Chem.

[48] Lu L, Li J, Moussaoui M, Boix E. Immune Modulation by Human Secreted RNases at the Extracellular Space. Front Immunol. 2018;9:1012. 10.3389/fimmu.2018.01012. 10.3389/fimmu.2018.01012 PMC5964141 29867984

[49] Jiang X, You L, Zhang Z, Cui X, Zhong H, Sun X (2021). Biological properties of Milk-derived extracellular vesicles and their physiological functions in infant. Front Cell Dev Biol.

[50] Eaton SA, Jayasooriah N, Buckland ME, Martin DI, Cropley JE, Suter CM (2015). Roll over Weismann: extracellular vesicles in the transgenerational transmission of environmental effects. Epigenomics..

[51] Do DN, Li R, Dudemaine PL, Ibeagha-Awemu EM. MicroRNA roles in signalling during lactation: an insight from differential expression, time course and pathway analyses of deep sequence data. Sci Rep. 2017, 7(October 2016):1–19. 10.1038/srep44605. 10.1038/srep44605 PMC5357959 28317898

[52] Wicik Z, Gajewska M, Majewska A, Walkiewicz D, Osińska E, Motyl T (2016). Characterization of microRNA profile in mammary tissue of dairy and beef breed heifers. J Anim Breed Genet.

[53] Luoreng ZM, Wang XP, Mei CG, Sen ZL (2018). Comparison of microRNA profiles between bovine mammary glands infected with Staphylococcus aureus and Escherichia coli. Int J Biol Sci.

[54] Rebane A, Akdis CA (2013). MicroRNAs: essential players in the regulation of inflammation. J Allergy Clin Immunol.

[55] Naeem A, Zhong K, Moisá SJ, Drackley JK, Moyes KM, Loor JJ (2012). Bioinformatics analysis of microRNA and putative target genes in bovine mammary tissue infected with streptococcus uberis. J Dairy Sci.

[56] Wang XP, Luoreng ZM, Sen ZL, Raza SHA, Li F, Li N (2016). Expression patterns of miR-146a and miR-146b in mastitis infected dairy cattle. Mol Cell Probes.

[57] Haneklaus M, Gerlic M, O’Neill LAJ, Masters SL (2013). MiR-223: infection, inflammation and cancer. J Intern Med.

[58] Jiao P, Wang X-P, Luoreng Z-M, Yang J, Jia L, Ma Y (2021). miR-223: an effective regulator of immune cell differentiation and inflammation. Int J Biol Sci.

[59] Ye D, Zhang T, Lou G, Liu Y. Role of miR-223 in the pathophysiology of liver diseases. Exp Mol Med. 2018;50(9). 10.1038/s12276-018-0153-7. 10.1038/s12276-018-0153-7 PMC6158210 30258086

[60] Han S, Li X, Liu J, Zou Z, Luo L, Wu R (2020). Bta-miR-223 targeting CBLB contributes to resistance to Staphylococcus aureus mastitis through the PI3K/AKT/NF-κB pathway. Front Vet Sci.

[61] Melnik BC, Schmitz G (2019). Exosomes of pasteurized milk: potential pathogens of Western diseases 06 biological sciences 0601 biochemistry and cell biology. J Transl Med.

[62] Shen B, Zhang L, Lian C, Lu C, Zhang Y, Pan Q, Yang R, Zhao Z. Deep Sequencing and Screening of Differentially Expressed MicroRNAs Related to Milk Fat Metabolism in Bovine Primary Mammary Epithelial Cells. Int J Mol Sci. 2016;17(2):200. 10.3390/ijms17020200. 10.3390/ijms17020200 PMC4783934 26901190

[63] Lawless N, Reinhardt TA, Bryan K, Baker M, Pesch B, Zimmerman D (2014). Microrna regulation of bovine monocyte inflammatory and metabolic networks in an in vivo infection model. G3 genes. Genomes, Genet.

[64] Lewandowska-Sabat AM, Hansen SF, Solberg TR, Østerås O, Heringstad B, Boysen P (2018). MicroRNA expression profiles of bovine monocyte-derived macrophages infected in vitro with two strains of Streptococcus agalactiae. BMC Genomics.

[65] Schanzenbach CI, Kirchner B, Ulbrich SE, Pfaffl MW (2017). Can milk cell or skim milk miRNAs be used as biomarkers for early pregnancy detection in cattle?. PLoS One.

[66] Jin W, Ibeagha-Awemu EM, Liang G, Beaudoin F, Zhao X, Guan L (2014). Transcriptome microRNA profiling of bovine mammary epithelial cells challenged with Escherichia coli or Staphylococcus aureus bacteria reveals pathogen directed microRNA expression profiles. BMC Genomics.

[67] Blankenberg D, Von Kuster G, Coraor N, Ananda G, Lazarus R, Mangan M, Nekrutenko A, Taylor J. Galaxy: a web-based genome analysis tool for experimentalists. Curr Protoc Mol Biol. 2010;Chapter 19:Unit 19.10.1-21. 10.1002/0471142727.mb1910s89. 10.1002/0471142727.mb1910s89 PMC4264107 20069535

[68] Bick JT, Flöter VL, Robinson MD, Bauersachs S, Ulbrich SE (2018). Small RNA-seq analysis of single porcine blastocysts revealed that maternal estradiol-17beta exposure does not affect miRNA isoform (isomiR) expression. BMC Genomics.

[69] Almiñana C, Tsikis G, Labas V, Uzbekov R, da Silveira JC, Bauersachs S (2018). Deciphering the oviductal extracellular vesicles content across the estrous cycle: implications for the gametes-oviduct interactions and the environment of the potential embryo. BMC Genomics.

[70] Kozomara A, Birgaoanu M, Griffiths-Jones S (2019). MiRBase: from microRNA sequences to function. Nucleic Acids Res.

[71] Robinson MD, McCarthy DJ, Smyth GK (2009). edgeR: a bioconductor package for differential expression analysis of digital gene expression data. Bioinformatics..

[72] Robinson MD, Oshlack A (2010). A scaling normalization method for differential expression analysis of RNA-seq data.

[73] Zhou X, Lindsay H, Robinson MD. Robustly detecting differential expression in RNA sequencing data using observation weights. Nucleic Acids Res. 2014;42(11):e91. 10.1093/nar/gku310. 10.1093/nar/gku310 PMC4066750 24753412

[74] Fischer DS, Theis FJ, Yosef N (2018). Impulse model-based differential expression analysis of time course sequencing data. Nucleic Acids Res.

[75] Licursi V, Conte F, Fiscon G, Paci P (2019). MIENTURNET: an interactive web tool for microRNA-target enrichment and network-based analysis. BMC Bioinformatics.

[76] Vlachos IS, Zagganas K, Paraskevopoulou MD, Georgakilas G, Karagkouni D, Vergoulis T (2015). DIANA-miRPath v3.0: deciphering microRNA function with experimental support. Nucleic Acids Res.

[77] Agarwal V, Bell GW, Nam JW, Bartel DP (2015). Predicting effective microRNA target sites in mammalian mRNAs. Elife..

