# Supplementary material for: Determining extracellular vesicles properties and miRNA cargo variability in bovine milk from healthy cows and cows undergoing subclinical mastitis
Source: BMC Genomics. 2022 Mar 7;23:189. doi: 10.1186/s12864-022-08377-z (PMC8903571; doi:10.1186/s12864-022-08377-z)
Supplement: Supplementary file 1 — Additional file 1: Supplementary Figure S1-S5. [file 12864_2022_8377_MOESM1_ESM.docx]

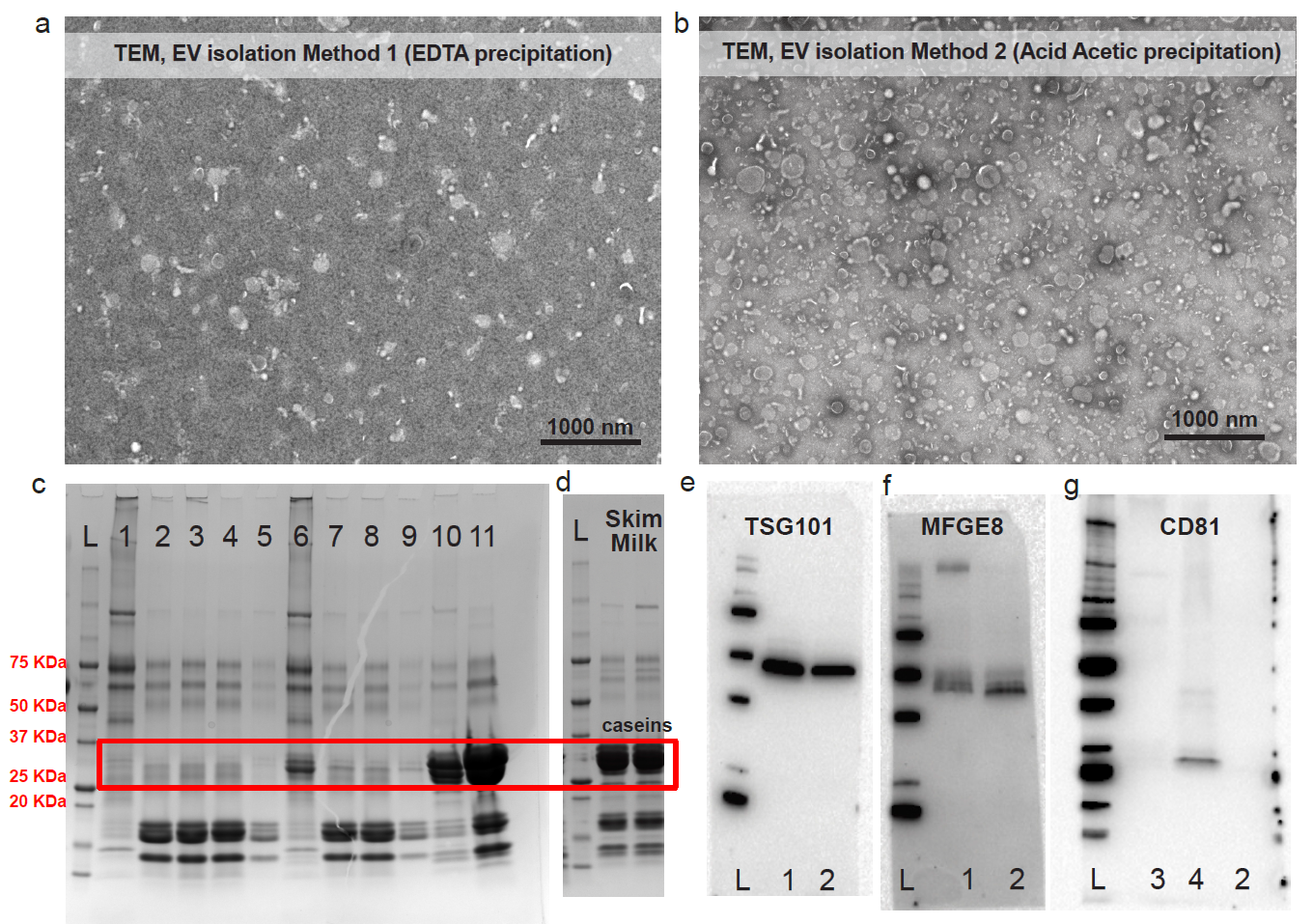


**Supplementary Figure S1.** Transmission electron microscopy (TEM) from EVs pellets obtained after a) Method 1 (EDTA 0.25M precipitation followed by differential ultracentrifugation 12’000g, 35’000g, 70’000g, 100’000g); b) Method 2 (Acid acetic 1% precipitation following 210’000g ultracentrifugation). c) SDS-PAGE protein profile of different samples stained with Blue Coomassie. L: ladder. 1: EVs pellet after Method 2 of isolation (described in detail in the Material and Methods section); 2: Supernatant from sample 1 after acid precipitation before 0.22 µm filtering. 3: Supernatant from sample 1 after 0.22 µm filtering; 4: Supernatant from sample 1 after the first round of 210’000g ultracentrifugation; 5: Supernatant from sample 1 after second round 210’000g ultracentrifugation; 6: EVs pellet after Method 2 of isolation with minor modification. The milk samples were stored in the freezer without 12’000g centrifugation 20 min; 7: Supernatant from sample 6 after acid precipitation before 0.22 µm filtering. 8: Supernatant from sample 6 after 0.22 µm filtering; 9: Supernatant from sample 6 after second round 210’000g ultracentrifugation; 10: EVs pellet after Method 1; 11: Supernatant after last round of centrifugation from sample 10. d) SDS-PAGE protein profile of skim milk samples stained with Blue Coomassie. e-g) Western blots of TSG101, MFGE8 and CD81. L: ladder; 1 and 2: EVs pellets after Method 1 isolation; 3 and 4: EV pellets after Method 2 isolation. During CD81 analysis, sample 3 had 10% β-Mercaptoethanol during the SDS-PAGE electrophoresis while samples 4 and 2 did not. Complete gels and blots are shown in Supplementary Figure S5.


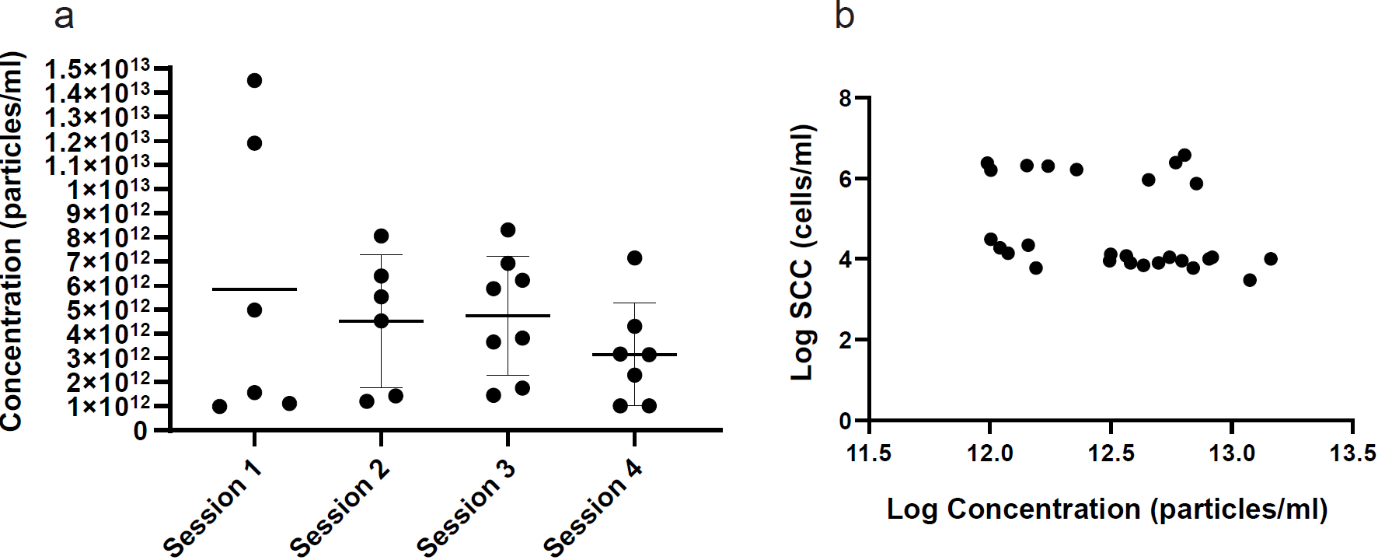


**Supplementary Figure S2.** Bioanalyzer profiles after RNA 6000 Pico assay (blue) and Small RNA assay (green). The extraction method and use or not of RNase treatment of EVs are mentioned above in the graphs.


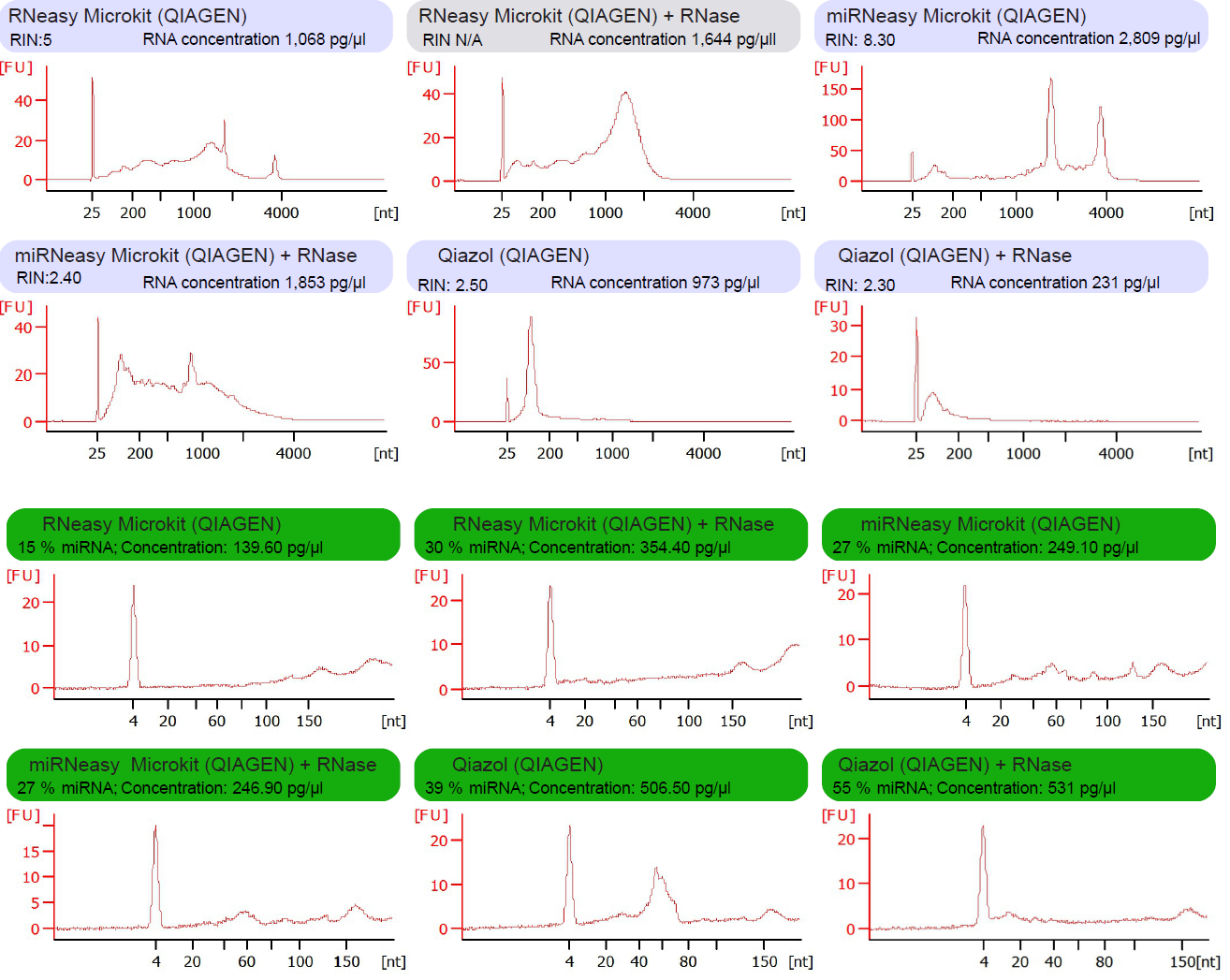


**Supplementary Figure S3.** a) EVs concentration obtained in each session of EVs isolation. b) Correlation between Somatic Cell Count (SCC) and EVs concentration, both in logarithmic scale.


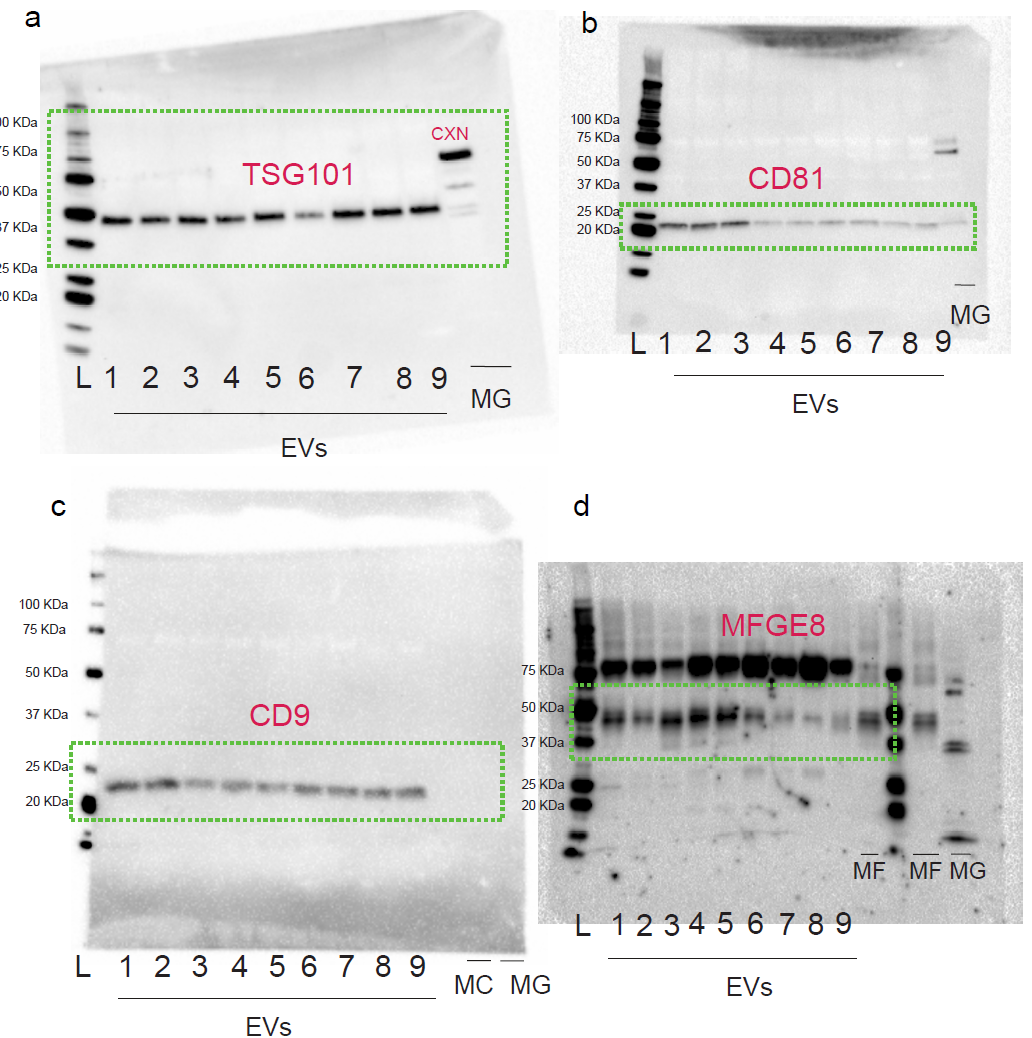


**Supplementary Figure S4.** a-d) Full-length western blot characterization for the EVs protein markers CXN, TSG101, CD81, CD9 and MFGE8. The green lines define the area shown in the main text. L: Ladder; 1-9: Milk EVs pellets. MG: Mammary gland tissue; MF: Milk Fat; MC: Milk cells.


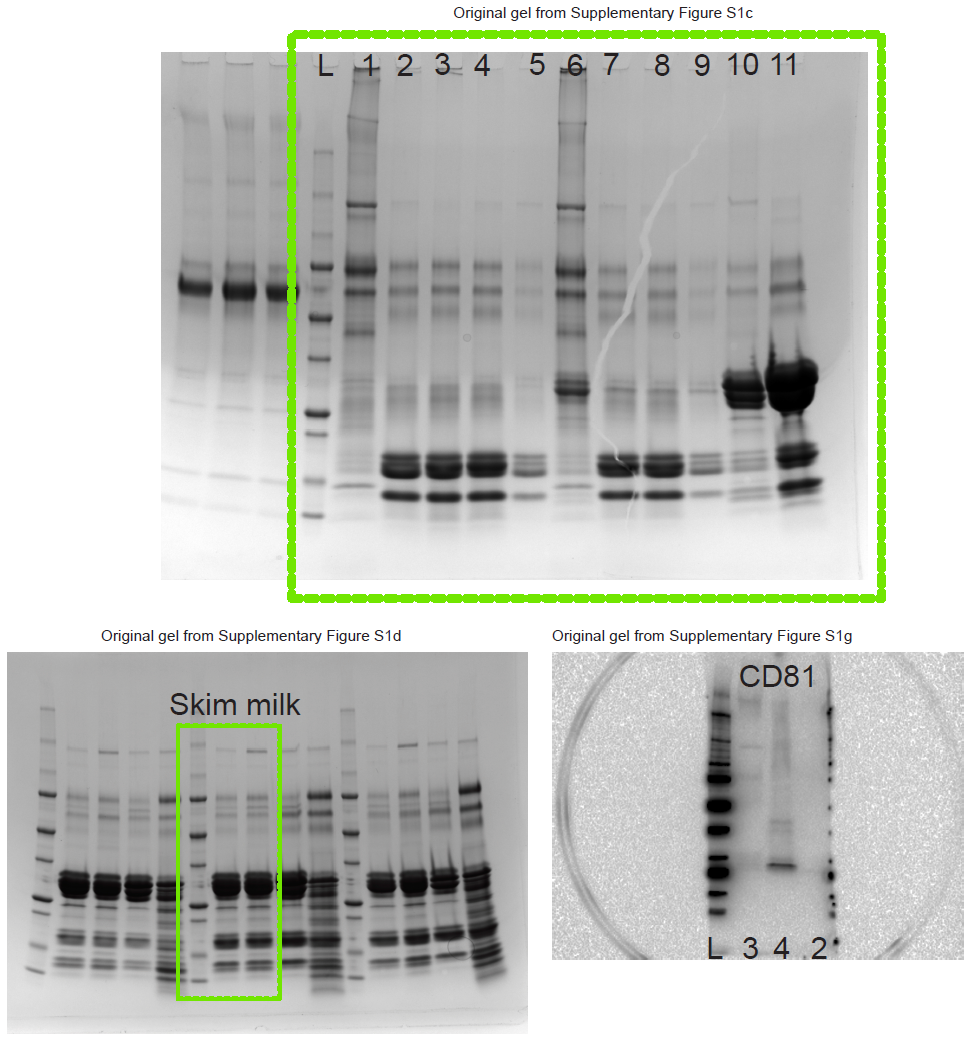


**Supplementary Figure S5.** Full-length gel and western blots from Supplementary Figure S1. The green lines define the area shown in Supplementary Figure S1.
