# Supplementary material for: Determining extracellular vesicles properties and miRNA cargo variability in bovine milk from healthy cows and cows undergoing subclinical mastitis
Source: BMC Genomics. 2022 Mar 7;23:189. doi: 10.1186/s12864-022-08377-z (PMC8903571; doi:10.1186/s12864-022-08377-z)
Supplement: Supplementary file 3 — Additional file 3: Supplementary Table S2. Mean size, mode size, concentration of particles and RNA concentration in each EV sample. [file 12864_2022_8377_MOESM3_ESM.docx]

**Supplementary Table S2.** Mean size, mode size and concentration of particles/ml in each EV sample evaluated with qNano (IZON). First number (C1-C6) indicates cow identity. Other abbreviations: C: Control group, L: Low SCC group, H: High SCC group, the number indicates sampling day (1,2 and 3, respectively).

| Sample | Mean (nm) | Mode (nm) | Min (nm) | Max (nm) | Concentration (particles/ml) | RNA (ng/μl) |
| --- | --- | --- | --- | --- | --- | --- |
| C1_C1 | 96 | 65 | 52 | 319 | 1.19E+13 | 2.1 |
| C1_C2 | 104 | 70 | 54 | 362 | 6.92E+12 | 1.4 |
| C1_C3 | 100 | 69 | 48 | 354 | 6.22E+12 | 1.4 |
| C2_C1 | 99 | 75 | 63 | 351 | 1.19E+12 | 9 |
| C2_C2 | 108 | 79 | 61 | 310 | 1.44E+12 | 4.8 |
| C2_C3 | 100 | 69 | 51 | 286 | 3.66E+12 | 3.6 |
| C3_C1 | 108 | 88 | 53 | 348 | 4.31E+12 | 0.9 |
| C3_C2 | 106 | 73 | 50 | 384 | 1.45E+13 | 3.2 |
| C3_C3 | 100 | 77 | 49 | 265 | 8.06E+12 | 1.1 |
| C4_H1 | 111 | 86 | 69 | 312 | 1.74E+12 | 1.6 |
| C4_H2 | 102 | 78 | 51 | 312 | 7.14E+12 | 1.6 |
| C4_H3 | 103 | 68 | 52 | 287 | 4.53E+12 | 1.7 |
| C5_H1 | 105 | 82 | 61 | 427 | 1.01E+12 | 1.6 |
| C5_H2 | 104 | 81 | 64 | 354 | 1.42E+12 | 1.6 |
| C5_H5 | 107 | 81 | 61 | 312 | 2.28E+12 | 2 |
| C6_H1 | 92 | 64 | 49 | 397 | 5.87E+12 | 1.4 |
| C6_H2 | 103 | 82 | 59 | 330 | 9.76E+11 | 2.1 |
| C6_H6 | 91 | 67 | 50 | 300 | 6.39E+12 | 1.8 |
| C4_L1 | 102 | 71 | 55 | 397 | 3.82E+12 | 1.4 |
| C4_L2 | 101 | 81 | 55 | 322 | 4.98E+12 | 1.9 |
| C4_L3 | 118 | 73 | 56 | 435 | 8.30E+12 | 1.9 |
| C5_L1 | 102 | 78 | 52 | 344 | 3.16E+12 | 1.3 |
| C5_L2 | 106 | 74 | 64 | 380 | 1.01E+12 | 0.62 |
| C5_L3 | 105 | 78 | 53 | 361 | 1.10E+12 | 3.1 |
| C6_L1 | 100 | 72 | 47 | 381 | 5.53E+12 | 0.93 |
| C6_L2 | 108 | 84 | 63 | 422 | 1.55E+12 | 1.1 |
| C6_L3 | 103 | 71 | 55 | 308 | 3.13E+12 | 0.81 |
