# Supplementary material for: Determining extracellular vesicles properties and miRNA cargo variability in bovine milk from healthy cows and cows undergoing subclinical mastitis
Source: BMC Genomics. 2022 Mar 7;23:189. doi: 10.1186/s12864-022-08377-z (PMC8903571; doi:10.1186/s12864-022-08377-z)
Supplement: Supplementary file 6 — Additional file 6: Supplementary Table S5. Top-ten most abundant miRNA in Control, Low SCC and High SCC samples. [file 12864_2022_8377_MOESM6_ESM.docx]

Supplementary Table S5.

| **Control** | Percentage (%) | **Low SCC** | Percentage (%) | **High SCC** | Percentage (%) |
| --- | --- | --- | --- | --- | --- |
| **bta-let-7a-5p** | 12.5 | **bta-let-7a-5p** | 15.2 | **bta-let-7a-5p** | 13.1 |
| **bta-miR-3596** | 8.1 | **bta-let-7b** | 8.6 | **bta-miR-3596** | 7.6 |
| **bta-let-7b** | 8.7 | **bta-miR-3596** | 9.4 | **bta-let-7b** | 8.1 |
| **bta-miR-200c** | 9.0 | **bta-miR-200c** | 10.1 | **bta-miR-21-5p** | 8.7 |
| **bta-miR-30a-5p** | 8.7 | **bta-miR-148a** | 9.1 | **bta-miR-200c** | 8.6 |
| **bta-miR-148a** | 8.9 | **bta-miR-30a-5p** | 8.6 | **bta-miR-148a** | 8.5 |
| **bta-miR-21-5p** | 8.6 | **bta-miR-26c** | 8.7 | **bta-miR-30a-5p** | 9.2 |
| **bta-miR-26c** | 9.3 | **bta-miR-21-5p** | 9.3 | **bta-let-7f** | 10.0 |
| **bta-let-7c** | 8.9 | **bta-let-7f** | 9.9 | **bta-miR-26c** | 8.6 |
| **bta-let-7f** | 8.5 | **bta-let-7c** | 10.9 | **bta-let-7c** | 8.3 |
