# Supplementary material for: Determining extracellular vesicles properties and miRNA cargo variability in bovine milk from healthy cows and cows undergoing subclinical mastitis
Source: BMC Genomics. 2022 Mar 7;23:189. doi: 10.1186/s12864-022-08377-z (PMC8903571; doi:10.1186/s12864-022-08377-z)
Supplement: Supplementary file 7 — Additional file 7: Supplementary Table S6. List of differential miRNAs between Control quarters of different cows. [file 12864_2022_8377_MOESM7_ESM.docx]

| Cow 2 vs. Cow 1 | | | |
| --- | --- | --- | --- |
| miRNA | Log2 FC | FDR | Cow 2 |
| bta-miR-2890 | 3.0 | 3.91E-03 | ↑ |
| bta-miR-196a | 1.9 | 1.91E-02 | ↑ |
| bta-miR-2904 | 1.5 | 4.59E-02 | ↑ |
| bta-miR-885 | 1.5 | 2.41E-02 | ↑ |
| bta-miR-181b | 1.1 | 4.68E-02 | ↑ |
| bta-miR-2898 | 1.0 | 3.62E-02 | ↑ |
| bta-miR-24-3p | -1.0 | 4.59E-02 | ↓ |
| bta-miR-3600 | -1.3 | 3.62E-02 | ↓ |
| bta-miR-143 | -1.8 | 3.92E-02 | ↓ |
| Cow 2 vs. Cow 3 | | | |
| miRNA | Log2 FC | FDR | Cow 2 |
| bta-miR-196a | 3.0 | 8.80E-04 | ↑ |
| bta-miR-2890 | 2.2 | 1.05E-02 | ↑ |
| bta-miR-11985 | 2.8 | 1.93E-02 | ↑ |
| bta-miR-3596 | 1.3 | 4.92E-02 | ↑ |
| bta-miR-885 | 1.1 | 4.92E-02 | ↑ |
| bta-miR-2898 | 0.8 | 4.92E-02 | ↑ |
| bta-miR-125a | 0.8 | 4.92E-02 | ↑ |
| bta-miR-24-3p | -1.0 | 4.92E-02 | ↓ |
| bta-miR-16a | -1.1 | 2.76E-02 | ↓ |
| bta-miR-3613a | -1.2 | 2.70E-02 | ↓ |

**Supplementary Table S6**. List of differential miRNAs between Control quarters of different cows (FDR<0.05). FC: Fold change; FDR: False Discovery Rate.
